# The Mucin Family of Proteins: Candidates as Potential Biomarkers for Colon Cancer

**DOI:** 10.3390/cancers15051491

**Published:** 2023-02-27

**Authors:** Kristin E. Cox, Shanglei Liu, Thinzar M. Lwin, Robert M. Hoffman, Surinder K. Batra, Michael Bouvet

**Affiliations:** 1 Department of Surgery, University of California San Diego, La Jolla, CA 92037, USA; 2 VA San Diego Healthcare System, La Jolla, CA 92161, USA; 3 Department of Surgical Oncology, City of Hope National Medical Center, Duarte, CA 91010, USA; 4 AntiCancer, Inc., San Diego, CA 92111, USA; 5 Department of Biochemistry and Molecular Biology, University of Nebraska Medical Center, Omaha, NE 68198, USA

**Keywords:** mucins, colorectal cancer, adenocarcinoma, mucinous carcinoma, hyperplastic polyps, adenomatous polyps, adenoma, serrated polyps, prognostics

## Abstract

**Simple Summary:**

Colorectal cancer is the second leading cause of cancer-related deaths in the United States with an overall 5-year survival of 65%. While there have been many advances in the treatment of this disease over the past few decades, there has been minimal change in the overall five-year survival in the past twenty years. Thus, there is still a need for the improved detection and treatment of this malignancy that affects many patients. Mucins are a family of glycoproteins (MUC1–MUC24) expressed by many epithelial tissues and some have been implicated in the progression of various malignancies. Mucins have diverse expression profiles amongst pre-malignant, malignant, and normal colonic tissues. This review article focuses on mucin expression profiles in normal and malignant colonic tissue as well as mucins’ role in diagnostics, therapeutics, and prognostication.

**Abstract:**

Mucins (MUC1–MUC24) are a family of glycoproteins involved in cell signaling and barrier protection. They have been implicated in the progression of numerous malignancies including gastric, pancreatic, ovarian, breast, and lung cancer. Mucins have also been extensively studied with respect to colorectal cancer. They have been found to have diverse expression profiles amongst the normal colon, benign hyperplastic polyps, pre-malignant polyps, and colon cancers. Those expressed in the normal colon include MUC2, MUC3, MUC4, MUC11, MUC12, MUC13, MUC15 (at low levels), and MUC21. Whereas MUC5, MUC6, MUC16, and MUC20 are absent from the normal colon and are expressed in colorectal cancers. MUC1, MUC2, MUC4, MUC5AC, and MUC6 are currently the most widely covered in the literature regarding their role in the progression from normal colonic tissue to cancer.

## 1. Introduction

Colorectal cancer (CRC) is the second leading cause of cancer-related deaths in the United States [1]. When detected early (T1), 5-year survival rates can be as high as 91%. Unfortunately, despite screening efforts, many colon cancers are still detected at advanced stages, making the overall 5-year survival 65% [2].

A series of well-defined malignant transformations has been described for the progression to CRC. These include the tubular adenoma (TA) and sessile serrated adenoma (SSA) pathways that form precancerous polyps and eventually progress to CRC [3,4,5,6,7]. TAs account for 65–70% of CRCs, while SSAs account for 15–30% of CRCs [8]. A hallmark of CRC is the inactivation of tumor suppressor genes such as adenomatous polyposis coli (APC), p53, and KRAS. Most CRCs (70–80%) possess a mutation in the APC gene, including both sporadic and germline mutations [9].

Mucins are a family of high-molecular-weight glycoproteins primarily synthesized by epithelial cells [10]. Mucins are characterized by tandem repeat structures and a high proportion of proline, threonine, and serine (the PTS domain) [11]. The human family of mucins consists of 24 members (MUC1 to MUC24), which can be subclassified into transmembrane or secreted mucins. Transmembrane mucins include MUC1, MUC3A/B, MUC4, MUC11-13, MUC15-17, MUC20, and MUC21. Secreted mucins include the gel-forming MUC2, MUC5AC/B, MUC6, and MUC19, and the non-gel-forming MUC7 [12,13] (Table 1).

The molecular mechanisms and signaling pathways of mucins are diverse. Transmembrane mucins contain EGF domains that allow them to participate in signal transduction [14]. The involved signaling pathways include MUC1 and TAK1, MUC1/MUC4 and WNT/β-catenin, MUC13 and ERK, and MUC16 and JAK/STAT [15,16,17]. They also play important roles in forming protective barriers, antigen presentation, and the production of antimicrobial peptides [14,18].

Mucins have been implicated in chronic inflammatory states and the promotion of oncogenesis in numerous malignancies including breast, lung, gastric, biliary, pancreatic, colorectal, and ovarian cancers. A large body of work has been generated since the first international meeting on “carcinoma-associated mucins” that was held in San Francisco in 1990 [19]. It is hypothesized that the abnormal expression of mucins disrupts cell–cell adhesions, thus facilitating tumor invasion [20]. For this review, we focus on mucins’ role in colorectal cancers, exploring the research behind their role in promoting tumorigenesis and how they can be used for diagnosis, prognostication, and potential treatment.

## 2. Methods

PubMed and Google Scholar were searched for publications on human mucins related to both normal colonic tissue and colon cancers published through January 2023. Inclusion criteria were as follows: (1) research concerning the investigation of human mucin proteins in colorectal cancer or their expression in the normal colon, (2) research with non-retracted findings, and (3) research accessible by the University of California, San Diego (UCSD) library. Exclusion criteria were as follows: (1) abstracts without published manuscripts available for review and (2) publications not available in English. For each mucin gene, the phrases “MUC#” OR “mucin#” AND “colon” OR “colorectal” were used as search terms (e.g., MUC3 colon). This returned 1356 entries, and each abstract was screened for possible inclusion; thus, 346 papers remained and were examined further. Upon reviewing their citations, an additional 9 papers were identified and a total of 129 papers were included in this review.

## 3. Results

### 3.1. Mucin 1

MUC1 is a large and highly glycosylated transmembrane mucin that was originally termed milk mucin, given its high expression in mammary glands [21,22,23]. It is known to have minimal or absent expression within normal colonic tissues (reported up to 10%), while it is upregulated in 54.5–100% of colorectal cancers (CRCs) [24,25,26,27,28,29,30,31]. In a 1994 study, Nakamori et al. showed that MUC1 expression increased with the advanced stage of the disease and when a tumor had metastasized [27]. Of the patients with Dukes stage C or D, 33.3% (7 of 21) had MUC1 levels five times that of normal tissue according to Western blotting, while 91.6% (11 of 12) of patients with Duke’s stage A or B had MUC1 expression levels less than two times that of normal tissue. Wang et al. observed MUC1 expression in 34.5% (9 of 26) of patients’ tumors without lymph node metastasis, while MUC1 expression was seen in 84.2% (16 of 19) of patients’ tumors with lymph node metastasis [28]. Within the signet ring subtype of CRC, 42% (*n* = 12) of cases were found to express MUC1 [32]. Baeckström et al. showed that the CRC cell lines Colo205 and SW1116 express MUC1, while LoVo did not express MUC1 [33]. Additionally, Devine et al. reported MUC1 to be expressed by HT29 [34]. Conflicting data have been reported on LS174T, with a slightly higher proportion of groups reporting negative MUC1 expression [33,34,35,36,37].

#### 3.1.1. MUC1 Mouse Studies

Mukherejee et al. demonstrated the ability of a MUC1 vaccine to prevent tumor growth in mice [38]. In the mice treated with a combination of MUC1 vaccine, granulocyte macrophage colony-stimulating factor (GM-CSF), and CpG motifs 7 days prior to injection with the CRC cell line MC38, all eight mice failed to grow tumors. When rechallenged with tumor cells two months later, the tumors again failed to grow. Suprunuik et al. studied the synergistic effects of platinum-based chemotherapeutics and anti-MUC1 antibodies in mouse models of colon cancer [39]. Using two human colon cancer cell lines, they studied the rates of apoptosis and changes in both mRNA and protein expression after treatment with each drug alone or in combination. The rate of apoptosis, as noted by Annexin V and Propidium iodine staining, increased from 13.7% to 30% when PtPz6 (a pyrazole-platinum complex) was combined with an anti-MUC1 monoclonal antibody. They further showed that the mRNA of the pro-survival proteins Bcl-xL and Bcl-2 was suppressed after treatment, while the pro-apoptotic factors Bax, Bad, Bim, and Bid were increased. One conflicting finding, however, was that while Bcl-xL mRNA was suppressed, protein expression was increased.

#### 3.1.2. Therapeutic Applications of MUC1

In 2006, Loveland et al. published a phase 1 clinical trial that utilized autologous dendritic cells treated with mannin-MUC1 fusion protein in ten patients with adenocarcinomas that were either stage IV or had progressed during prior therapy. They included patients with colorectal, esophageal, lung, ovarian, fallopian, breast, and renal cell cancers. They showed that this treatment elicited an IFN**γ**-mediated T-cell response in all patients, among which a patient with CRC had stable disease for 7 months, and two patients (breast and renal CA) were able to achieve 3-year remission with subsequent dendritic cell immunotherapy [40]. When the study progressed to phase 2 testing among ovarian cancer patients, a statistically significant improvement in progression free survival and overall survival was seen in the subset that had required second-line therapies to achieve remission [41,42]. Karanikas et al. attempted direct immunization with a mannin-MUC1 fusion protein in patients with primarily breast or colorectal cancer, though only 20% demonstrated cellular immunity following vaccination [43,44].

More recently, a phase 1 trial of an anti-MUC1 monoclonal antibody was completed in 2013 [45]. Multiple MUC1-expressing tumors were analyzed in the study, including colon (33.5%), ovarian (27%), breast (9.5%), non-small cell lung cancer (9.5%), and pancreatic cancer (6.8%). Unfortunately, when the tested antibody, Gatipotuzumab, was taken to phase 2 testing in ovarian cancer, no benefit was seen compared to the placebo [46]. A phase 2 trial of another MUC1 vaccine is currently underway to evaluate its ability to decrease recurrence rates in patients with a history of advanced adenoma (those with high-grade dysplasia, villous/tubulovillous features, or tumors larger than 1 cm) [47].

Another therapeutic use of MUC1 was explored by affixing MUC1 aptamers to exosomes containing doxorubicin for selective drug delivery. While in vitro studies did not show an improvement over doxorubicin alone, when implemented in in vivo mouse models, the tumor volume growth rate was significantly reduced. Additionally, all mice survived to 30 days compared to only two of the five mice surviving when treated with doxorubicin containing exosomes alone, while none of the control mice survived [48].

#### 3.1.3. Prognostication with MUC1

Li et al. performed a meta-analysis of 16 studies and found that high MUC1 expression was associated with worse overall survival (HR 1.51) (95% CI 1.30–1.75, *p*-value < 0.00001). Additionally, high MUC1 expression was associated with a higher stage (RR 1.44), depth of invasion (RR 1.30), and lymph node metastasis (RR 1.47) [49].

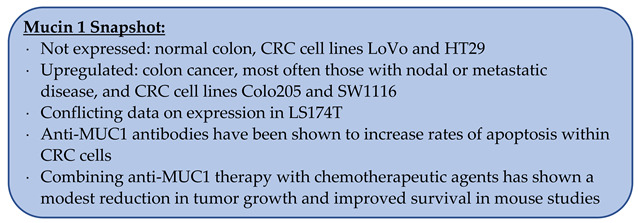



### 3.2. Mucin 2

**Figure 1:**
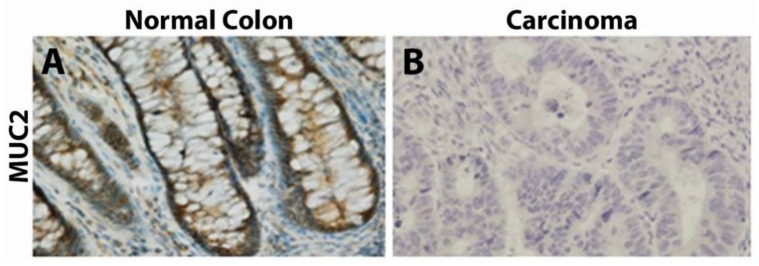
Immunohistochemistry of MUC2 from Wang et al., *Oncology Letters* 2017, reprinted with permission [56]. (**A**) Cytoplasmic MUC2 staining in normal colon. (**B**) Minimal to no staining within carcinoma. Magnification 200x.

MUC2 is part of the secreted and gel-forming subset within the mucin family that is synthesized and secreted by goblet cells [50]. The intestinal epithelium is covered by a thick layer of mucus for protection, of which MUC2 is a major component [51]. MUC2 has been shown to be expressed in normal colonic tissue, while its decreased expression is associated with non-mucinous colon adenocarcinomas [52,53,54,55,56] (Figure 1). Although decreased MUC2 expression is associated with colorectal adenocarcinoma, its expression is always maintained in mucinous carcinomas [57]. A large study of 702 patients conducted by Walsh et al. revealed that 33% of the analyzed tumors expressed MUC2 [58]. Bu et al. found similar results, with MUC2 expression seen in 46.2% of colorectal adenocarcinoma (*n* = 26), 100% of mucinous carcinoma (*n* = 15), and 87.5% of signet-ring cell carcinoma (*n* = 8) [59].

#### 3.2.1. MUC2 Expression within Polyps

MUC2 expression is maintained in hyperplastic polyps, sessile serrated polyps, and traditional serrated adenomas [60]. This pattern is not surprising, as normal colonic tissue expresses MUC2; therefore, these polyps have not yet lost their expression the way some non-mucinous adenocarcinomas have. Using multivariate analysis, Krishn et al. were able to demonstrate that the loss of MUC2 was a significant predictor of adenoma/adenocarcinoma vs. hyperplastic polyps [61].

#### 3.2.2. MUC2 Mouse Studies

In mice deficient in Muc2, colonic inflammation and superficial erosions are seen that mimic ulcerative colitis as early as 5 weeks old [62]. When allowed to survive for 6 months, they develop adenomas; at 1 year, the majority have progressed to adenocarcinomas [63].

#### 3.2.3. Prognostication with MUC2

Cecchini et al. evaluated multiple biomarkers’ ability to predict prognosis in stage II colon cancer [64]. They focused on stage II, as prior studies noted variable prognosis within this stage and sought to identify a subgroup for which a survival benefit might be found for adjuvant chemotherapy. They evaluated 210 cases of stage II colon cancer and found that the complete loss of MUC2 expression resulted in a hazard ratio of 3.32 (95% CI 1.20–9.20).

Low levels of MUC2 expression have also been shown to correlate with lymph node metastasis [56]. Wang et al. evaluated low-MUC2- and high-MUC2-expressing colon tumors and found that 52.8% (38 of 72) of low-MUC2-expressing tumors had lymph node metastasis compared to 35.8% (24 of 67) of high-MUC2-expressing tumors (*p*-value < 0.05). Additionally, they showed a significantly reduced 5-year survival for low-MUC2-expressing tumors of 40.2% compared to 73.9% for high-MUC2-expressing tumors [56]. In a meta-analysis, Li et al. found low MUC2 expression corresponded to a worse overall survival with an HR of 1.67 [65].

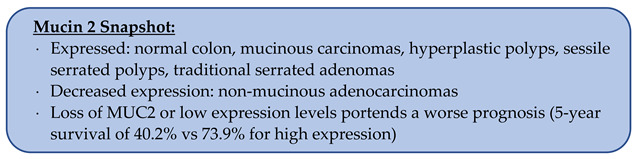



### 3.3. Mucin 3

MUC3, part of the membrane-bound subset within the mucin family, has two discrete yet very similar MUC3 genes (MUC3A and MUC3B) [66,67]. In addition to MUC1, MUC2, and MUC4, MUC3 is expressed in normal colonic tissue [52,68,69]. Throughout the body, the highest expression of MUC3 is seen in the duodenum [70]. Williams et al. evaluated ten CRC cell lines (Caco-2, LIM1215, LIM1899, HCT116, SW116, LoVo, LS174T, KM12SM, LISP-1, and SW620) and found that all lines except SW620 expressed MUC3 according to RT-PCR data [71]. However, when Gum et al. performed similar experiments, LS174T only expressed MUC3 (by RNA blot) when cultured in the presence of Butyrate (the main energy source of intestinal cells) [70]. When comparing the MUC3 expression levels in colon cancers to normal tissue, lower levels were seen in CRCs via immunohistochemistry and in situ hybridization [55].

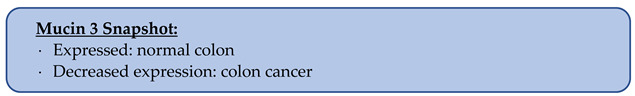



### 3.4. Mucin 4

**Figure 2:**
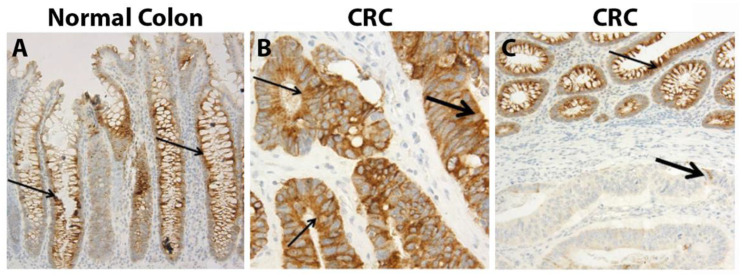
IHC of MUC4 from Shanmugam et al., *Cancer* 2010, reprinted with permission [78]. (**A**) MUC4 staining was noted in crypts of the normal colon. (**B**) Strong cytoplasmic staining within CRC. (**C**) Weak staining of CRC compared to adjacent normal tissue. Thin arrows—normal colonic epithelium; thick arrows—CRC. Scale: (**A**) 200 μm; (**B**) 600 μm; (**C**) 200 μm.

MUC4, a transmembrane mucin protein, is normally expressed in respiratory and colonic epithelial cells [68,70,72]. Abnormal expression of MUC4 has been implicated in breast [73], ovarian [74], lung [75], gallbladder [76], and biliary malignancies [77]. Regarding CRC, there have been conflicting reports as to whether MUC4 is overexpressed or lost. Shanmugam et al. characterized 132 CRCs and found that 25% (*n* = 33) had high MUC4 expression while 6% (*n* = 8) had a complete loss of MUC4 expression [78] (Figure 2). However, Krishn et al. reported undetectable levels of MUC4 in 63% of colorectal adenocarcinomas, a large difference from the 6% loss of MUC4 that Shanmugam et al. reported, though only 16 samples were evaluated [61].

#### 3.4.1. MUC4 Expression within Polyps

Krishn et al. evaluated 10 hyperplastic polyps and 30 adenomatous polyps, in which the majority were found to have lower expression of MUC4 compared to normal tissue, and only 13% of adenomas had strong expression [61].

In a study by Biemer-Hüttmann et al., hyperplastic polyps showed a reduction in MUC4 expression, with 6 of 12 completely negative for MUC4 and 4 showing reduced staining patterns. No change in the expression of tubular adenomas was seen compared to normal tissue, while serrated adenomas showed a complete loss of MUC4 [79].

#### 3.4.2. MUC4 Mouse Studies

In Muc4 knockout mouse studies (*Muc4^-/-^*), Muc2 mRNA was significantly increased compared to wild-type (WT) mice. When inflammation was induced with dextran sodium sulfate (DDS), Muc3 was also found to increase. Following DDS treatment, these *Muc4^-/-^* mice had improved survival compared to the wild type. Interestingly, both the *Muc4^-/-^* and WT female mice had improved survival compared to their male counterparts (survival at 21 days: *Muc4^-/-^* female 100%, WT female 30%, *Muc4^-/-^* male 10%, and WT male 0%) [80].

**Figure 3:**
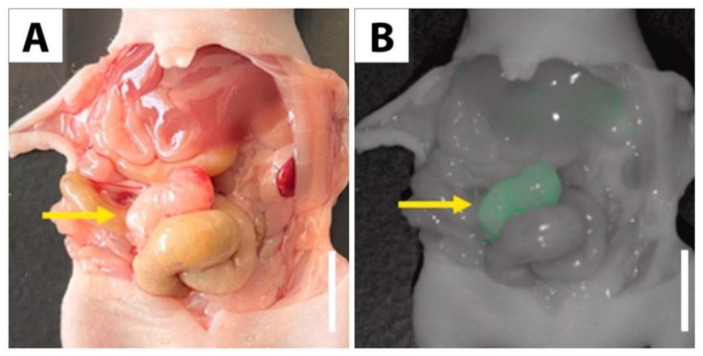
Orthotopic model of LS174T labeled with MUC4-IR800 reported by Turner et al., *The American Journal of Surgery* 2022, reprinted with permission [81]. (**A**) Bright light image of an LS174T tumor growing from the cecum. (**B**) Fluorescence imaging of MUC4-IR800 brightly labelling a cecal tumor. Yellow arrows: tumor. Scale: 1 cm.

In orthotopic mouse models of CRC, MUC4 conjugated to a near-infrared dye (IR800) effectively targeted and labeled primary colorectal tumors and liver metastasis (Figure 3). Both a cancer cell line (LS174T) and a patient-derived tumor were used in these experiments. In vivo imaging was performed, and tumor-to-background ratios (TBR) of ~2 were observed for primary tumors, while TBRs of 1.56 were observed for liver metastasis [81].

#### 3.4.3. Prognostication with MUC4

MUC4 mutation has been shown to be an independent predictor of survival among CRC patients. Peng et al. evaluated the tumor mutational burden of over seven hundred samples from The Cancer Genome Atlas (TCGA) and the International Cancer Genome Consortium (ICGC) and found 17 genes that were commonly mutated amongst both groups (*APC*, *TP53*, *TNN*, *KRAS*, *MUC16*, *MUC4*, *SYNE1*, *FLG*, *FAT4*, *OBSCN*, *FAT3*, *RYR2*, *PIK3CA*, *FBXW7*, *DNAH11*, *MUC5B*, and *ZFHX4*). MUC4 mutation was the only gene associated with a significantly worse survival compared to wild type MUC4 for the patients in the TCGA (*p*-value of 0.009) [82].

Shanmugam et al. reported shorter disease-free survival for high-MUC4-expressing CRCs, with a hazard ratio of 2.07 (95% CI 1.14–3.75, *p*-value of 0.017). This shortened survival was most pronounced in early-stage (I and II) CRC with a hazard ratio of 3.77 (95% CI 1.46–9.73, *p*-value of 0.006) [78]. Additionally, in patients without distant metastasis, two single nucleotide polymorphisms (SNPs) of the MUC4 gene, rs3107764 and rs842225, were associated with differential overall survival and event-free survival [83]. Lu et al. demonstrated that the substitution of GG for CC at this SNP resulted in a reduction in 5-year survival from ~78% to ~42% [83].

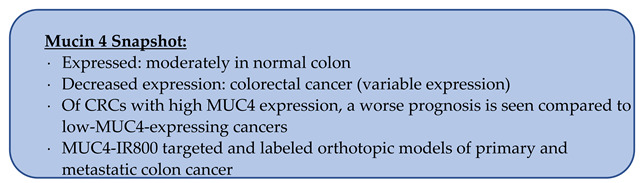



### 3.5. Mucin 5

MUC5AC and MUC5B are secreted, gel-forming mucin proteins. Both MUC5AC and MUC5B are absent (or have been found to be present at extremely low levels) in normal colonic tissue [52,61,84]. MUC5AC expression has been noted in many CRCs, most often in mucinous carcinomas [53]. A large study of 702 patients in Melbourne revealed that 50% of the tumors expressed MUC5AC, and 53% expressed MUC5B [58]. MUC5AC expression has also been reported in the HT29 and SW620 CRC cell lines [85]. When evaluating histopathologic subtypes of CRCs, Imai et al. found that 30.2% of well-to-moderately differentiated CRCs (*n* = 63) expressed MUC5AC while 51.6% of poorly differentiated CRCs (*n* = 91) expressed MUC5AC [86].

#### 3.5.1. MUC5 Expression within Polyps

**Figure 4:**
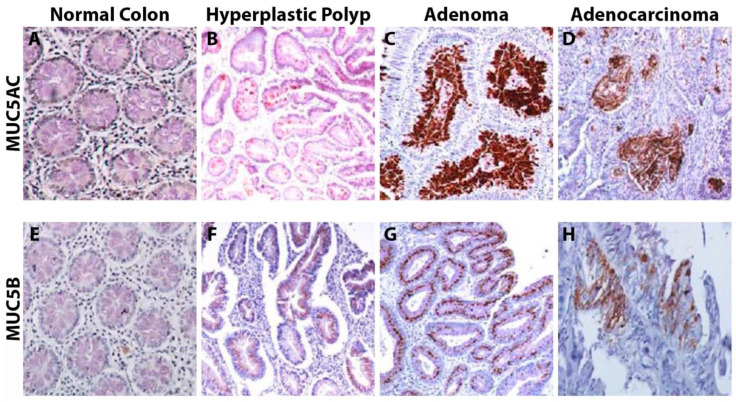
IHC of MUC5AC and MUC5B from Krishn et al., *Cancer Letters* 2016, reprinted with permission [61]. (**A**) No MUC5AC staining was present within the normal colon. (**B**–**D**) Increased MUC5AC expression was seen in hyperplastic polyps, adenomas, and adenocarcinoma. (**E**) No MUC5B staining was seen within the normal colon. (**F**–**H**) Rare instances of MUC5B expression were seen in hyperplastic polyps, adenomas, and adenocarcinoma.

Numerous groups have reported varying percentages for MUC5AC expression amongst different subtypes of colonic polyps (Table 2, Figure 4). For hyperplastic polyps, these values have ranged from 11–100%, while traditional and sessile serrated adenomas (TSA and SSA) ranged from 31–43% and 61–100%, respectively [60,84,87].

An interesting subclass that Bartman et al. evaluated was that of adenomas with villous features. Of the 120 colonic polyps they evaluated, which ranged from 0.5 cm to greater than 2 cm, adenomas with villous features had a higher percentage of MUC5AC expression at 40.7% (*n* = 86) compared to that of 24% for tubular (*n* = 25) [84].

Kim et al. also further examined polyp subgroups, evaluating adenomas with low-grade vs. high-grade dysplasia, though no difference in MUC5AC expression was observed (12% for low-grade vs. 10% for high-grade). However, when evaluating 175 microsatellite-unstable (MSI-H) CRCs, of which 76 were sporadic and 99 were associated with lynch syndrome, MUC5AC-positive tumors were significantly associated with sporadic tumors (54% vs. 27% for lynch syndrome; *p*-value < 0.001). Another significant difference was observed regarding the location of the tumor. Of the MUC5AC-positive tumors, 46% were proximal compared to 25% that were distal (*p*-value of 0.005) [87].

#### 3.5.2. Tumor Imaging with MUC5AC

MUC5AC has been used for detecting xenografts of human colon cancer lines via MRI. Rossez et al. screened peptides against MUC5AC for positive selection and MUC2 for negative selection, given the latter’s abundant expression in normal colonic tissue. By attaching the best peptide (the peptide with the highest affinity to MUC5AC and lowest or no affinity to MUC2) to small iron oxide particles, they could visualize tumors with MRI. They confirmed its specificity for labeling MUC5AC-producing tumors by using the HT29 cell line as a positive control and HCT116 as a negative control [89].

#### 3.5.3. Prognostication with MUC5AC

High-MUC5AC-expressing tumors have been shown to more commonly be poorly differentiated [56,86]. Additionally, Wang et al. demonstrated that these tumors were more likely to have higher rates of lymph node metastasis (*p*-value < 0.01) and higher tumor stages (*p*-value < 0.01). The 5-year survival was also significantly reduced for high-MUC5AC-expressing tumors at 29.8% compared to 69.9% for low-MUC5AC-expressing tumors (*p*-value < 0.001) [56].

While poorly differentiated CRCs have a worse prognosis than well-to-moderately differentiated CRCs, and are more likely to express MUC5AC, a lack of MUC5AC expression amongst poorly differentiated CRCs indicates a worse prognosis. Recurrence-free 5-survival was ~53% vs 20% for high- vs. low-MUC5AC-expressing tumors (*p*-value of 0.004). Overall survival showed a similar trend, though it was non-significant, with a *p*-value of 0.1 [86].

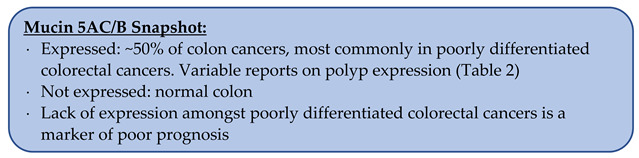



### 3.6. Mucin 6

MUC6 is a member of the gel-forming secreted subset of mucins. It was originally called gastric mucin when it was first described in 1992 by Toribara et al. due to its high expression within the stomach [90]. MUC6 has minimal to no expression within normal colonic tissue [68,84]. Reports on MUC6 expression within CRCs range from 18.8–39% [58,61,68]. In the signet-ring cell subtype, 0% (*n* = 12) were found to express MUC6 [32]. Amongst microsatellite-unstable CRCs, Kim et al. reported that 13.7% (24 of 175) expressed MUC6 protein, and no significant difference was seen between sporadic and hereditary non-polyposis colorectal cancer [87]. High levels of MUC6 expression have been reported in the CRC cell line LS174T [91].

#### MUC6 Expression within Polyps

Numerous groups have reported varying percentages for MUC6 expression amongst different subtypes of colonic polyps, with values ranging from 0–16.9% for hyperplastic polyps, 0–16% for adenomas, and 20–100% for sessile serrated adenomas (SSA) (Table 3) [60,61,68,84,87,92,93]. A few of these studies noted significant differences in MUC6 expression based on the proximal vs. distal location of the polyps. Fujita et al. noted that 60% of proximal HPs expressed MUC6 compared to only 4% of distal polyps (*p*-value < 0.0001) [60]. Within SSAs, IHC revealed that 94.1% of proximal polyps expressed MUC6 compared to only 26.9% of distal polyps [93].

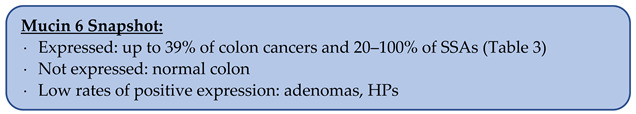



### 3.7. Mucins 7 and 8

MUC7 and MUC8 expression have not been observed within the small bowel or colon [52].

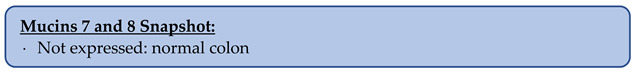



### 3.8. Mucin 11

MUC11 is a member of the transmembrane subtype of mucins. It was first described in 1999 by Williams et al. [94]. Via Northern blotting, they were able to show that MUC11 was expressed in normal colon samples and was either absent or significantly reduced in paired CRC samples. They also evaluated MUC11 mRNA expression in multiple CRC cell lines and found that HT29, LIM1215, LIM1899, and LIM1863 had very faint expression, while SW620 and SW480 had relatively high levels of MUC11 expression. In patient-derived tumor samples, 80% (12 of 15) had down-regulated MUC11 levels compared to paired normal colon samples.

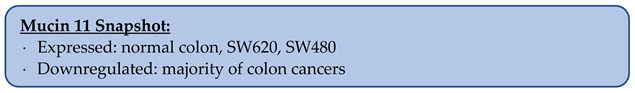



### 3.9. Mucin 12

MUC12 is a transmembrane mucin that is expressed within the normal colon and weakly in the pancreas [94]. Multiple studies have shown the downregulation of MUC12 mRNA in some CRCs compared to normal colons [94,95,96,97]. Although, some CRCs still express relatively high levels of MUC12. Pham at el. found that 62.9% (39 of 62) of CRCs expressed MUC12, of which 35.9% (*n* = 14) had weak or mild staining and 64.1% (*n* = 25) had intense staining [98]. When only evaluating the 37 CRCs with metastatic disease, similar ratios were found for the staining patterns (13.5% weak/mild and 40.5% intense). When present in CRC, MUC12 localization is altered, with apical staining observed in normal colons and a loss of polarity seen in CRCs [98]. Additionally, Williams et al. found that MUC12 mRNA was not expressed in the colon cancer cell lines HT29, LIM1215, LIM1899, LIM1863, SW620, or SW480 [94].

#### Prognostication with MUC12

Matsuyama et al. evaluated the MUC12 mRNA expression levels in 73 patients with stage II or III CRC and found that the 3-year disease-free survival was reduced to 66.1% for patients with low-MUC12-expressing tumors compared to 90.9% for those with high-MUC12-expressing tumors (*p*-value of 0.02). Worse survival for low-MUC12-expressing tumors of any stage was also demonstrated by Wu et al. [99]. Upon multivariate analysis, tumor MUC12 expression levels were found to be an independent prognostic factor [100].

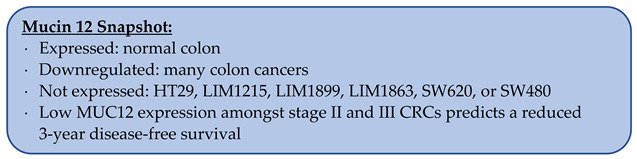



### 3.10. Mucin 13

MUC13 is a transmembrane mucin that is cleaved into two subunits and then undergoes homodimerization [101]. It has been shown to be expressed in the normal colon to varying degrees and is confined to the apical membrane [101,102,103]. There have been discrepancies regarding MUC13 expression in CRC; Packer et al. and Williams et al. both reported a downregulation of MUC13 mRNA levels [95,101], while Walsh et al. and Gupta et al. reported MUC13 expression equal to or greater than paired normal samples in 100% of tumors evaluated by IHC [102,103]. Additionally, Gupta et al. demonstrated that aberrant localization of MUC13 staining (i.e., expression on the basal surface, in the cytoplasm, or in the nucleus) was more commonly seen in metastatic CRCs. Cytoplasmic staining was seen in 23.7% of non-metastatic tumors compared to 89.3% for metastatic disease. Nuclear staining was observed in 10.5% of non-metastatic tumors and 64.3% of metastatic tumors. Variable expression has been observed within colon cancer cell lines. SW620, LoVo, T-84, and HT-29 had detectable MUC13 RNA levels, while these were faint or absent from SW48 and SW480 [103]. LIM2463, LS513, SW116, and SW620 were found to have high levels of MUC13 mRNA [91,101].

In subsequent work, Gupta et al. further characterized the role of MUC13 in tumorigenesis by creating CRC cell lines that had exogenous MUC13 expression (by transfecting a GFP-tagged MUC13 vector) or knock-down expression (with a shRNA lentivirus). The MUC13-overexpressing line showed statistically significant increases in cell growth, the ability to form colonies, and cell migration compared to the control. Importantly, the inverse was true for the MUC13 knock-down cell line, wherein a significant reduction was seen in all three tumorigenic features [104].

#### 3.10.1. MUC13 Mouse Studies

Using a Muc13 knockout mouse (*Muc13^-/-^*) and a colitis-associated colorectal (CAC) tumorigenesis model induced by AOM and followed by DDS, Sheng et al. showed that *Muc13^-/-^* mice had fewer and smaller tumors and decreased hyperplasia compared to wild-type CAC models [105]. They also found that the anti-apoptotic protein Bcl-xL was upregulated in WT mice but not in *Muc13^-/-^* mice, suggesting that Muc13 was important for the prevention of apoptosis. However, when Bcl-xL was blocked, no change in the number of tumors was seen in the WT or *Muc13^-/-^* mice, which conflicted with the prior hypothesis.

#### 3.10.2. Prognostication with MUC13

Sheng et al. evaluated 88 cases of CRC and found that low MUC13 expression (by IHC) predicted a significantly reduced 5-year survival of 45% (*n* = 60) compared to 90% (*n* = 28) for high-MUC13-expressing tumors (*p*-value of 0.0006) [105]. However, Sojka et al. found improved survival with low MUC13 expression (*n* = 187) [106].

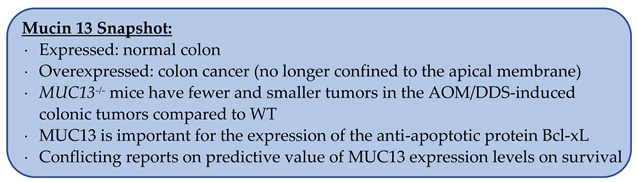



### 3.11. Mucin 14

MUC14 is a transmembrane mucin protein that has been scarcely researched with respect to its expression profiles or roles in human tissues. In a genomic analysis, Reynolds et al. reported a higher rate of MUC14 mutations in microsatellite-stable mucinous CRC (4.44%) compared to non-mucinous CRC (0.24%) [107].

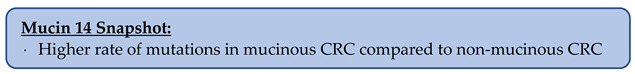



### 3.12. Mucin 15

MUC15 is a transmembrane mucin of ~100–120 kDa that was first described by Pallesen et al. in 2002 [108]. Low levels of MUC15 mRNA have been reported in normal colonic tissue, while many CRCs exhibit overexpression (sometimes as high as 10-fold compared to matched normal tissue) [108,109]. Huang et al. found that 70.8% (51 of 72) of patient-derived CRCs had MUC15 overexpression (as determined by RT-PCR) while 82.7% (43 of 52) had overexpression determined via IHC [109]. MUC15 has also been shown to be more highly expressed in poorly differentiated CRC compared to well- or moderately differentiated CRC [110].

Huang et al. also utilized the transfection of MUC15 vectors into HCT116 (a low-MUC15-expressing CRC cell line) with or without shRNA to study its effects on cell proliferation, apoptosis, and tumor growth. They found that MUC15 overexpression led to a statistically significant increase in cell proliferation, which was blocked by treatment with shRNA; however, no effect on apoptosis was seen [109].

#### MUC15 Mouse Studies

When the HCT116 cells transfected with MUC15 vectors were implanted subcutaneously in mice, the tumors were significantly larger (with a six-fold higher weight) compared to tumors grown from cells transfected with a mock vector. Additionally, Ki67 expression was significantly increased in the MUC15-positive cells compared to the control (74.3% versus 38.0%, *p*-value of 0.01), indicating increased cell proliferation [109].

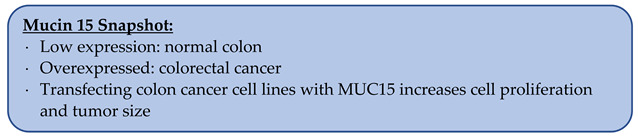



### 3.13. Mucin 16

**Figure 5:**
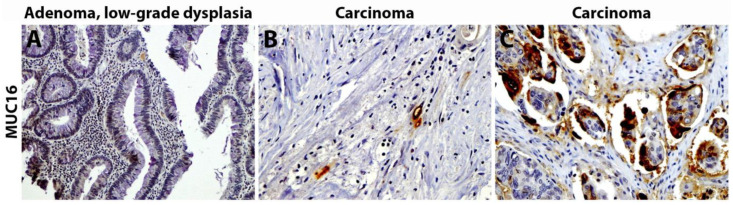
IHC of MUC16 and MUC16 from Streppel et al., *Human Pathology* 2012, reprinted with permission [111]. (**A**) No MUC16 staining was seen in an adenoma with low-grade dysplasia. (**B**) Focal and (**C**) Diffuse MUC16 staining within carcinoma samples. Magnification: (**A**) 10x; (**B**,**C**) 20x.

MUC16 is the largest of the transmembrane mucins and is clinically known as cancer antigen 125 (CA125) [111]. Streppel et al. demonstrated that MUC16 is not expressed within normal colonic tissue, while 64.1% (25 of 39) of CRCs express MUC16 (Figure 5) [111]. A significant difference in the rates of MUC16 expression between right-sided CRCs (23%, *n* = 206) and left-sided CRCs (9.8%, *n* = 214) was observed by Ward et al. [112]. A significant difference was also seen in the MUC16 expression levels between stages A–B and stages C–D, with lower expression levels seen in the earlier stages of CRC (*p*-value of 0.037) [113]. Liu et al. also demonstrated that elevated levels of MUC16 mRNA can be detected in the peripheral blood of patients with CRC compared to healthy individuals [114].

Huang et al. investigated the predictive value of MUC16 serum levels towards the presence of peritoneal disease compared to CEA [115]. They found that MUC16 had improved specificity (89.2%) compared to CEA (62.8%), though its sensitivity was lower at 61.4% for MUC16 vs. 75.4% for CEA (*p*-value < 0.01). However, no significant difference in MUC16 levels was seen with increasing stages except for stage IV with peritoneal dissemination (PD). Interestingly, CEA levels predicted stage IV disease, although only if PD was absent [115].

#### Prognostication with MUC16

Multiple groups have investigated the prognostic value of MUC16 expression towards CRC, the majority of which found a significantly worse prognosis for those with elevated serum MUC16 levels (Table 4 and Table 5). Giessen-Jung et al. did not find a statistically significant difference in 5-year survival based on MUC16 expression; however, they excluded patients with metastatic disease and patients who received neoadjuvant therapy [116]. Streppel et al. evaluated CRCs via IHC and found that absent MUC16 expression (*n* = 14) had significantly worse mean survival compared to CRCs with focal staining (*n* = 15), namely, 87.3 months (95% CI 34.0–140.5) vs. 182.6 months (95% CI 143.1–222.1), respectively [111].

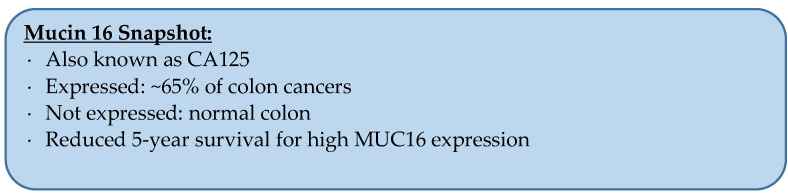



### 3.14. Mucin 17

MUC17 is a transmembrane mucin that is expressed in the normal colon and small intestine [61,70]. MUC17 is downregulated in inflammatory states such as ulcerative colitis and ischemic colitis [118]. Wolff et al. evaluated 148 CRC samples for mutations in 38 genes of interest and found that 21.6% (32 of 148) of CRCs had a MUC17 mutation [119]. Additionally, the human colon cancer cell line LS174T has consistently been shown to express MUC17 [70,118,120].

MUC17 has been shown to play a role in cell adhesion, adherence, and migration. Luu et al. used siRNA to MUC17 to silence its expression in LS174T cells. They saw reduced adhesion (96.25% vs. 91.67%; *p*-value < 0.002), reduced aggregation, and 67% less migration (*p*-value < 0.0001) compared to the controls. Additionally, when cells were treated with etoposide, those treated with MUC17 siRNA showed a significant increase in rates of apoptosis (6.52% vs. 1.75%; *p*-value < 0.002) [120].

#### 3.14.1. MUC17 Expression within Polyps

Krishn et al. showed that 60% of adenomas (*n* = 30) had strong MUC17 expression while hyperplastic polyps (*n* = 10) showed similar staining levels to those of the normal colon [61]. The work by Delker et al. also highlighted MUC17 expression as a distinguisher between sessile serrated adenomas and hyperplastic polyps as they found an 82-fold increase in MUC17 RNA expression in SSAs compared to HPs [121].

#### 3.14.2. MUC17 Mouse Studies

In mouse models of induced colitis (using acetic acid or dextran sodium sulfate), Luu et al. reported a significant reduction in crypt damage scores and degrees of ulceration for mice treated with exogenous MUC17 compared to the controls [120].

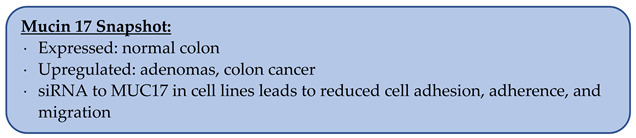



### 3.15. Mucin 18

MUC18 is a membrane-bound, mucin-like protein that is also known as CD146 and the melanoma cell adhesion molecule (MCAM). MUC18 is not expressed within the normal colonic mucosa [122]. Tian et al. found that 20% (*n* = 1080) of CRC samples expressed MUC18. Additionally, a higher proportion of MUC18 expression was seen in those with liver metastasis (39.2%, *n* = 102) vs. those without liver metastasis (18%, *n* = 978) [123]. Liu et al. also reported MUC18 expression in the CRC cell lines HT29 and SW948; this expression was absent from the SW480, SW620, and Colo205 lines [122].

#### MUC18 Mouse Studies

In knockdown xenograft models of MUC18 using CRC cell lines transfected with MUC18 shRNA, tumors lacking MUC18 grew faster than the controls (tumors visible at ~20 days compared to ~36 days) [122].

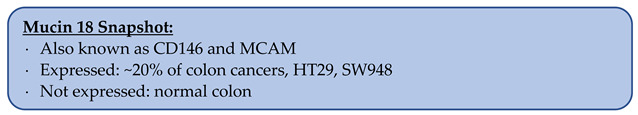



### 3.16. Mucin 19

MUC19 is a secreted mucin that has been limitedly researched with regard to colorectal cancer. However, amongst CRC lung metastasis samples, MUC19 mutations have been identified that are not present in the primary tumor, thus suggesting a role played by MUC19 concerning distant spread [124].

### 3.17. Mucin 20

MUC20 is a membrane-bound mucin for which minimal research has been conducted compared to other mucins. Xiao et al. is the only group to publish research on MUC20 and its role in CRC [125]. They reported MUC20 expression in 61.7% (91 of 150) of CRC and only 12% (18 of 150) of adjacent normal colon cells (*p*-value < 0.05). They also transfected CRC cell lines (LoVo and SW620) with GFP-shRNA-MUC20 or GFP-MUC20 to silence or express MUC20, respectively. They reported significantly reduced cell migration in the cells transfected with the shRNA; the opposite was seen in the GFP-MUC20-transfected cells.

#### Prognostication with MUC20

Xiao et al. reported MUC20 overexpression correlated with both increased recurrence and death [125]. Of the 47 patients with recurrence, 76.6% (*n* = 36) had MUC20-expressing tumors, while only 23.4% (*n* = 11) had tumors lacking MUC20 (*p*-value of 0.016). The rates of MUC20 positivity were similar in the patients without recurrence (55.6% MUC20+ and 44.4% MUC20-). Of the 41 deaths, 78% (*n* = 32) had MUC20-positive tumors, while 21.9% (*n* = 9) did not express MUC20 (*p*-value of 0.015).

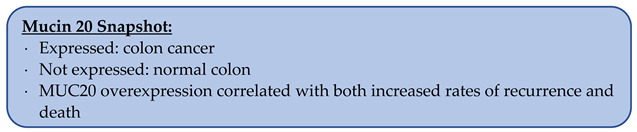



### 3.18. Mucin 21

MUC21 is a transmembrane mucin that Ito et al. first described in 2007 [126]. They reported MUC21 mRNA expression in the lung, thymus, and colon. However, when King et al. evaluated mRNA expression, they found MUC21 was absent from the normal colon and that its expression increased with the increasing stage of CRC [127].

#### Prognostication with MUC21

Vymetalkova et al. evaluated microRNA binding site polymorphisms in multiple mucin genes and their relation to colorectal cancer [128]. They reported that after adjusting for sex, age, smoking status, and cancer stage, there was a statistically significant reduction in overall survival for the CC genotype of rs886403 in MUC21 (HR 2.63, 95% CI 1.69–4.10, and *p*-value < 0.0001).

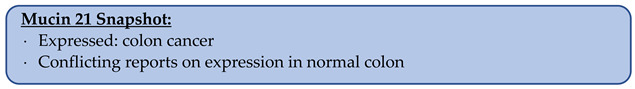



## 4. Discussion

Mucins are a family of glycoproteins containing 24 members that play diverse roles in cell signaling, barrier protection, and cell migration in many organ systems. They have been implicated in chronic inflammatory states and the promotion of oncogenesis in numerous malignancies, including breast [73], lung [75], gastric [11,129], biliary [76,77], pancreatic [11,111], colon, and ovarian cancers [46,74]. It is hypothesized that they aid tumor invasion by disrupting cell–cell adhesions [13].

While there is variability amongst colorectal cancers, general expression patterns have been established for many of the mucins. Those found to be expressed in normal colons include MUC2, MUC3, MUC4, MUC11, MUC12, MUC13, and MUC15 (at low levels) [52,53,54,55,68,70,94,101,102,103,108,109]. Those absent from the normal colon, and, subsequentially, found to be aberrantly expressed in CRC, include MUC5, MUC6, MUC16, and MUC20 [52,61,68,84,111,121]. This group of mucins that is absent from normal colons, yet is abnormally expressed in colorectal cancer, is a potential target for continued research into imaging studies geared towards improved detection or the delivery of therapeutic agents. Unfortunately, of these four genes, the highest percentage of positivity within CRCs was only 61.7% for MUC20.

Many mucins were also shown to be independent predictors of prognosis (Table 6), with worse prognosis for the low expression of MUC2, MUC12, and MUC13 [65,100,105] and the high expression of MUC4, MUC5, MUC16, and MUC20 [56,78,86,112,113,116,117,121].

When evaluating new biomarkers for colon cancer, MUC1, MUC2, MUC4, MUC5AC, and MUC6 are currently documented in the largest bodies of work regarding their role in the progression from normal colonic tissue to malignancy. Of these mucins, MUC6 seems promising in terms of its role as a biomarker of colorectal cancer, given its lack of expression in normal tissue and the relative consensus on its polyp expression profile (specifically, its expression is absent from benign hyperplastic polyps yet present in sessile serrated adenomas). However, MUC6 expression within CRC is lower (up to 39%) compared to other mucins such as MUC1, which is expressed in 84% of patients with lymph node metastases [28,68]. Thus, a combined approach utilizing multiple mucin proteins might be the next step for the specific labeling of pre-malignant and malignant tumors or for the targeted delivery of therapeutics.

## 5. Conclusions

In this review, we provided an overview of the current data on the mucin expression profiles in normal colons, benign and pre-malignant polyps, and colon cancer. While there have been some variations in these expression patterns, trends regarding the presence or absence of mucins in various tissue types can be ascertained. This includes the absence of MUC5, MUC6, MUC16, and MUC20 from the normal colon; maintained MUC2 expression in polyps; aberrant MUC5AC expression in sessile serrated adenomas; and the lack of MUC6 expression in benign hyperplastic polyps. Overall, there has been extensive research into the roles mucins play in the progression of colorectal cancer, and this work provides promising material for future developments in mucin-related diagnostics or therapeutics.

## Figures and Tables

**Table 1:** The Mucin family of proteins.

Gene	Cytogenetic Band	Form
MUC1	1q22	Membrane-bound mucin
MUC2	11p15.5	Secreted
MUC3	7q22	Membrane-bound mucin
MUC4	3q29	Membrane-bound mucin
MUC5AC	11p15.5	Secreted
MUC5B	11p15.5	Secreted
MUC6	11p15.5	Secreted
MUC7	4q13.3	Secreted
MUC8	12q24.33	Secreted
MUC9	1p13.2	Secreted
MUC12	7q22.1	Membrane-bound mucin
MUC13	3q21.2	Membrane-bound mucin
MUC14	4q24	Membrane-bound mucin
MUC15	11p14.2	Membrane-bound mucin
MUC16	19p13.2	Membrane-bound mucin
MUC17	7q22.1	Membrane-bound mucin
MUC18	11q23.3	Membrane-bound mucin
MUC19	12q12	Secreted
MUC20	3q29	Membrane-bound mucin
MUC21	6p21.33	Membrane-bound mucin
MUC22	6p21.33	Membrane-bound mucin
MUC24	6q21	Membrane-bound mucin

**Table 2:** MUC5AC expression within polyps.

Normal Colon	HP	Adenoma	TSA	SSA	Study
-	75.4% (49/65)	-	43.1% (31/72)	80.4% (41/51)	Fujita et al. [60]
0% (0/9)	20% (2/10)	27% (8/30)	-	-	Krishn et al. [61]
10.7% (3/28)	100% (8/8)	100% (19/19)	-	100% (10/10)	Perçinel et al. [68]
0% (0/26)	11% (1/9)	24% (6/25)	-	-	Bartman et al. [84]
0% (0/18)	43.4% (23/53)	-	31% (5/16)	61% (19/31)	Kim et al. [87]
-	15.2% (5/33)	8.3% (3/36)	-	61.5% (24/39)	Krishn et al. [88]

HP = Hyperplastic polyp, TSA = Tubular serrated adenoma, and SSA = Sessile serrated adenoma.

**Table 3:** MUC6 expression within polyps.

HP	Adenoma	SSA	Study
16.9% (11/65)	-	39.2% (20/51)	Fujita et al. [60]
-	10% (1/10)	-	Krishn et al. [61]
-	15.8% (3/19)	20% (2/10)	Perçinel et al. [68]
0% (0/9)	16% (4/25)	-	Bartman et al. [84]
4.3% (1/23)	0% (0/63)	51.6% (16/31)	Kim et al. [87]
0% (0/48)	-	100% (26/26)	Owens et al. [92]
17.4% (16/92)	0% (0/87)	53.5% (23/43)	Bartley et al. [93]

HP = Hyperplastic polyp, SSA = Sessile serrated adenoma.

**Table 4:** MUC16’s effect on 5-year survival.

Study	Sample Size	5-Year Survival	*p*-Value
Björkman et al. 2019 [113]	148	75.3% (low expression) vs. 50.7% (high expression)	<0.001
Giessen-Jung et al. [116]	472	87.1% (elevated MUC16) vs. 84.9% (overall)	0.3114
Björkman et al. 2020 [117]	282	66.7% (low expression) vs. 41.1% (high expression)	<0.001

**Table 5:** MUC16 Hazard Ratios.

Study	Sample Size	Hazard Ratio	95% Confidence Interval	*p*-Value
Ward et al. [112]	420	2.06	1.51–8.14	0.011
Björkman et al. 2019 [113]	148	1.91	1.45–2.53	<0.001
Björkman et al. 2020 [117]	282	2.48	1.68–3.65	<0.001

**Table 6:** Hazard ratios for overall survival based on variable expression levels of mucins.

Mucin and Level	Subset of CRC	HR	95% CI	*p*-Value	Study
High MUC1		1.51	1.30–1.75	<0.00001	Li et al. [49]
Loss of MUC2	Stage II	3.32	1.20–9.20	0.021	Cecchini et al. [64]
Low MUC2		1.67	1.43–1.94	<0.00001	Li et al. [65]
High MUC4		2.07	1.14–3.75	0.017	Shanmugam et al. [78]
High MUC4	Stage I and II	3.77	1.46–9.73	0.006	Shanmugam et al. [78]
High MUC16		2.06	1.51–8.14	0.011	Ward et al. [112]
High MUC16		1.91	1.45–2.53	<0.001	Björkman et al. [113]
High MUC16		2.48	1.68–3.65	<0.001	Björkman et al. [117]
